# An investigation of using log‐file analysis for automated patient‐specific quality assurance in MRgRT

**DOI:** 10.1002/acm2.13361

**Published:** 2021-07-18

**Authors:** Seng Boh Lim, Paola Godoy Scripes, Mary Napolitano, Ergys Subashi, Neelam Tyagi, Laura Cervino Arriba, Dale Michael Lovelock

**Affiliations:** ^1^ Memorial Sloan Kettering Cancer Center New York NY USA; ^2^ Standard Imaging Inc. Middleton WI USA

**Keywords:** elekta unity, linacview, log‐file analysis, MRI guided adaptive radiotherapy, MRgRT, patient specific quality assurance

## Abstract

**Objective:**

Adaptive radiation therapy (ART) is an integral part of MR‐guided RT (MRgRT), requiring a new RT plan for each treatment fraction and resulting in a significant increase in patient‐specific quality assurance (PSQA). This study investigates the possibility of using treatment log‐file for automated PSQA.

**Method:**

All treatment plans were delivered in 1.5T Unity MR‐Linac (Elekta). A Unity compatible version of LinacView (Standard Imaging) was commissioned to automatically monitor and analyze the log‐files. A total of 220 fields were delivered and measured by ArcCheck^®^‐MR (Sun Nuclear) and LinacView. Thirty incorrectly matched fields were also delivered to check for error detection sensitivity. The gamma analysis, γ, with 3%, 3 mm criteria was used in both ArcCheck^®^‐MR and LinacView. Additionally, the gantry angle, jaws, and multileaf collimators (MLC) positions reported in the log‐file were compared with plan positions using TG‐142 criteria.

**Result:**

The γ (3%, 3 mm) for the 190 plans were found to be between the range of 72.5%–100.0% and 95.4%–100.0% for ArcCheck^®^‐MR and LinacVeiw, respectively. All the delivered gantry angle and jaws were found to be within 0.2° and 2 mm. MLCs that were outside the guard leaves or under the diaphragms were found to have more than 1.0 mm discrepancy. This was attributed to the linac internal override for these MLCs and had no dosimetric impact. Excluding these discrepancies, all MLC positions were found to be within 1.0 mm. The γ (3%, 3 mm) for the 30 incorrectly matched fields were found to be 3.9%–84.8% and 0.1%–64.4% for ArcCheck^®^‐MR and LinacVeiw, respectively.

**Conclusion:**

Significant ranked correlation demonstrates the automated log‐file analysis can be used for PSQA and expedite the ART workflow. Ongoing PSQA will be compared with log‐file analysis to investigate the longer term reproducibility and correlation.

## 1 INTRODUCTION

Interfractional anatomical change in patients remains a challenge in radiation therapy. Adaptive radiation therapy (ART) has been shown to be effective in improving the target coverage and reducing normal tissue toxicity.^1^ The novel 1.5T magnetic resonance imaging‐based linear accelerator (MR‐LINAC), Unity Elekta, is an MR‐guided radiation therapy (MRgRT) treatment machine. The superior soft tissue definition of the MR T2 imaging allows more accurate tissue delineation resulting in potential toxicity reduction.^1^, ^2^ As the online ART is part of the standard clinical workflow of the 1.5T MRgRT, new plans, tailored to the patients' inter‐fractional anatomical changes, are generated to provide more accurate dosimetry and delivery.^1^

However, this online ART workflow results in a significant increase in patient‐specific quality assurance (PSQA). For a typical 30 fraction prostate plan, the workload of PSQA alone will increase by 30 times. A more efficient method to QA each adapted plan is essential to manage the otherwise overwhelming PSQA workload. Modern radiation therapy machines record the treatment delivery, such as delivered monitor units, multileaf, and gantry positions, in the machine log‐files at a high temporal frequency. This enables log‐file analysis to perform more granular analysis than typical detector‐based PSQA^3^, ^4^ and capture errors that may otherwise be undetected.^5^, ^6^ With the high demand of PSQA and the novelty of the machine, the benefit of such QA tool to complement the detector‐based tools cannot be understated.

Recently, Menten et al^7^ developed an in‐house solution to utilize the log‐files generated by the machine to QA the treatment delivery. However, as of the writing of this study, there is no commercial product that can automatically perform log‐file analysis for the 1.5T Unity MR‐Linac. It is hypothesized that the log‐files can accurately monitor the treatment delivery and can be used for PSQA. We investigated the feasibility of using the log‐files to automate PSQA process and expedite the current ART workflow.

## 2 METHOD

All treatment plans were delivered with a 1.5T Unity MR‐Linac (Elekta). A Unity compatible version of LinacView (Standard Imaging) was commissioned to automatically monitor and analyze the log‐files. A total of 190 fields, based on hypo‐fractionated rectum, prostate, and gastrointestinal plans, were delivered and measured by ArcCheck^®^‐MR (Sun Nuclear) and LinacView. Basing on the RT plan and log‐file information, LinacView generated fluences for comparison. The ability to detect incorrect plan delivery was assessed by analyzing 30 mismatched fields. These were adapted plans for the same patient but taken from a different day. The gamma anlaysis,^8^ γ, with 3%, 3 mm criteria with global normalization and 10% low dose threshold was used in both ArcCheck^®^‐MR and LinacView. The γ passing scores of ArcCheck and log‐file based were represented as γ_AC_ and γ_LV_. A set of γ passing thresholds, γ_t_, of 90.0%, 92.5%, 95.0%, and 97.5% were applied to each pair of γ_AC_ and γ_LV_. If the passing score of a datapoint fell above γ_t_, it was assigned to “1.” Otherwise, a “0” was assigned. Using the measurement‐based system as the ground truth, a 2 × 2 contingency analysis^9^ was performed for each pair of data to calculate the significance of the association between as γ_AC_ and γ_LV,_ with the contingency coefficient ϕ, and the corresponding p‐value. In this analysis, the parameter of ϕ can be interpreted as the usual Pearson's correlation coefficient^10^ with the values ranging from −1.0 to +1.0. Here, the ϕ values of −1, 0, and +1 correspond to negative association, no association, and positive association, respectively between the log‐file analysis and measurements. Table 1 shows an example of a 2 × 2 contingency table used in this study.

**TABLE 1:** An example of the 2 × 2 contingency table used in this study where a, b, c, and d are the frequency of each category

γ metric Threshold	3%, 3 mm 90%	ArcCheck	
		Fail	Pass
LinacView	Pass	*a*	*b*
	Fail	*c*	*d*

These contingency coefficients were used to assess the sensitivity and association strength between the two systems. An additional set of data with tighter tolerance of (3%, 2 mm) was also calculated to evaluate the effect on the association and sensitivity. Additionally, the gantry angle, jaws, and multileaf collimators (MLC) positions reported in the log‐file were compared with plan positions using TG‐142 criteria.^11^

**FIGURE 1:**
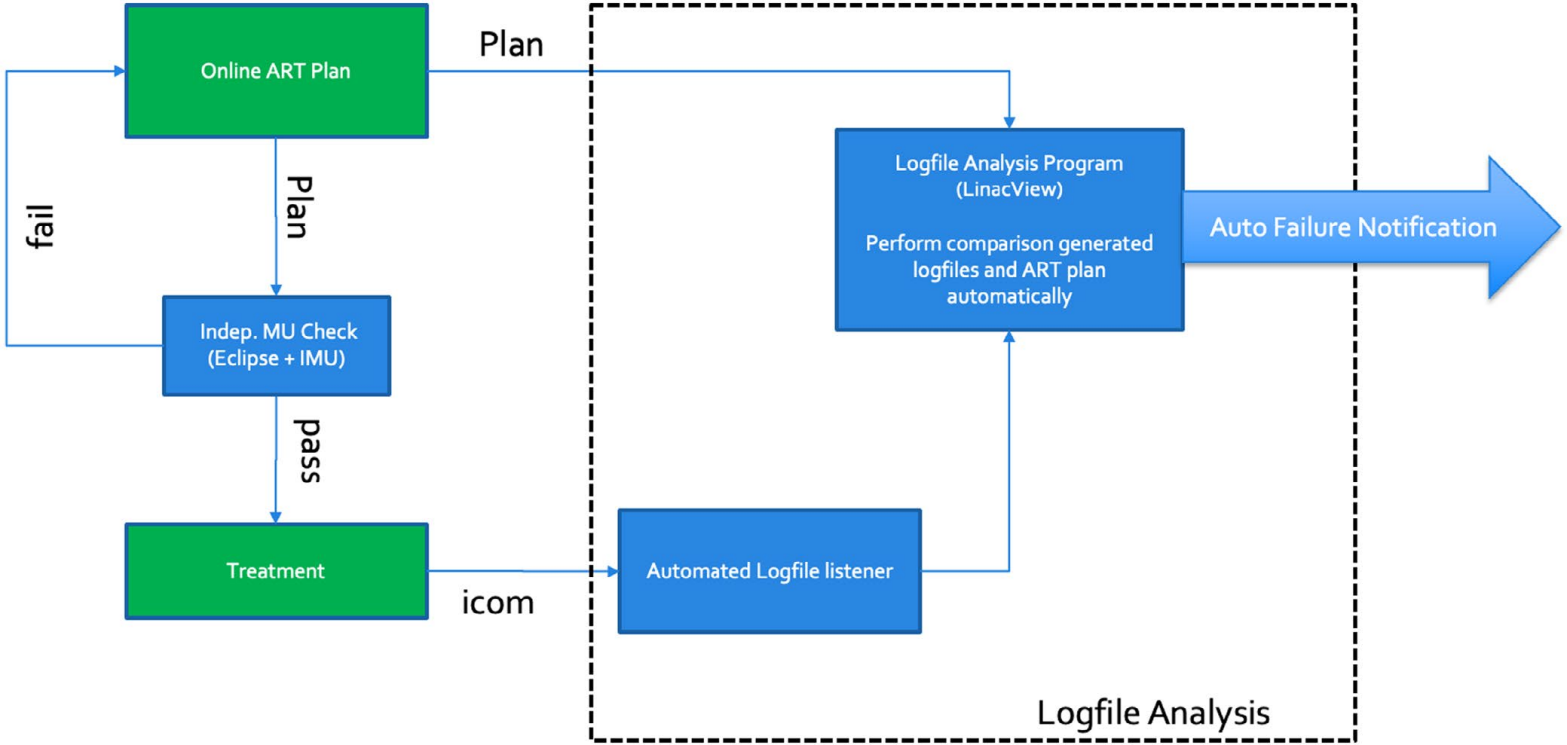
A schematic of the log‐file analysis (in dotted box) that interfaces with the Unity online planning system

Figure 1 shows the schematic of the daily treatment workflow and the corresponding log‐file analysis for the Unity. After the daily MR was performed for a patient, the patient remained in treatment position while an online adaptive plan was being generated. Here, Monaco treatment planning system (MTPS) (Elekta), a Monte Carlo dose calculation, was used as the primary dose calculation algorithm. Similar to the recent experience by Snyder,^12^ the MTPS of this study^13^ was commissioned using MPPG 5a criteria.^14^ As soon as a radiation therapy (RT) plan was completed, it was sent to a secondary dose calculation engine, Eclipse Independent MU check program^15^ (Eclipse+IMU), which used the Analytical Anisotropic Algorithm in the Eclipse planning system (Varian) to generate a dose distribution and compared it with that from Monaco. The treatment would proceed when Eclipse+IMU passed. In addition, pretreatment PSQA, based on the baseline plans, and the posttreatment PSQA, based on the daily plans, were performed with ArcCheck^®^.

As the MR‐Linac and the dedicated management systems were protected behind a network security services firewall (NSS), special arrangements were made to monitor log‐files from outside the NSS. The linear accelerator verification interface (iCOM‐VX), the communication between the machine controller and the MR‐LINAC, was modified to allow third party monitoring. This traffic was then forwarded to a special port at the NSS that can be accessed by LogfileSync (Standard Imaging), an independent automated log‐file listener. Once the listener finished recording the delivery of each treatment, a log‐file would be generated and sent to the log‐file analysis program. The listener recorded the MU, time, gantry, and collimator angles, couch, MLC, and diaphragms positions at a sampling rate of 4 Hz.

During the on‐line adaptation, the RT plan would also be exported to the predetermined location where the log‐file analysis program constantly monitors for import. Once the program received a RT plan and the corresponding log‐file, it could perform the analysis immediately after the delivery of each field during a typical multi‐field treatment. The program was also set up to send out automated warning electronic mail to designated parties if the analysis failed to meet the predetermined criteria.

## 3 RESULTS

The iCom‐VX instruction set of the MR‐Linac was found to be slightly different from the standard Elekta machine rendering the MLC positions not recorded properly. The library set of the log‐file listener was updated and modified to fully decode the communication. Four test fields were delivered to test the functionality of the log‐file listener. The accuracy of the independently generated log‐files by LogfileSync was confirmed with the internal logs by the MR‐Linac.

**FIGURE 2:**
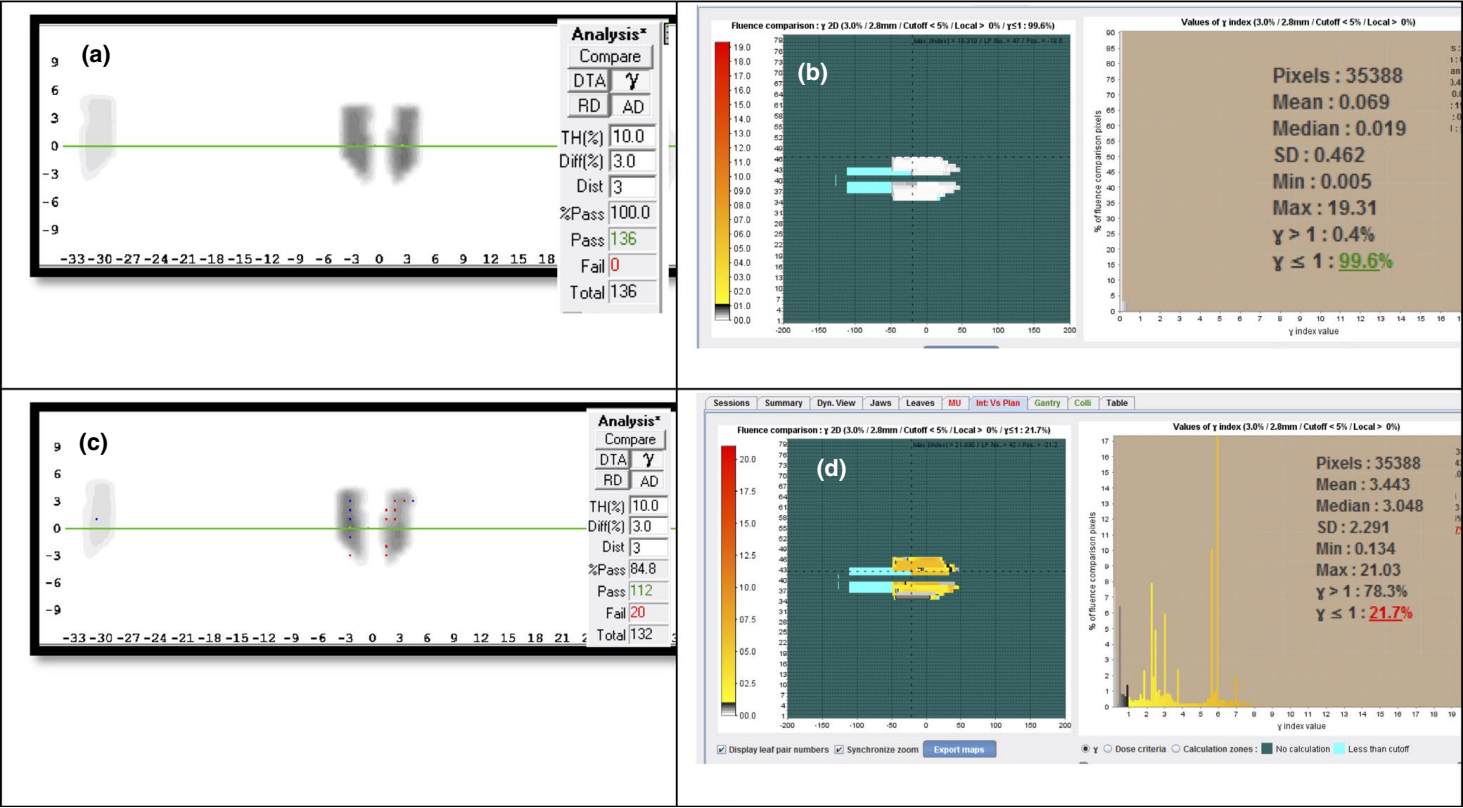
(A) ArcCheck®‐MR analysis and (B) log‐file fluence analysis from LinacView of a typical delivery with comparable (100% and 99.6%) γ passing rate; Incorrect delivered plan measured with (C) ArcCheck®‐MR and (D) LinacView with significant lower passing rate of 84.8% and 21.7%

The accuracy of the gantry and jaw reported by LinacView was found to be within 0.2° and 2 mm compared to the measurements by a high precision digital level and the on‐board imager, respectively. The log‐file‐based gamma analysis was found to give similar results to that reported by the ArcCheck^®^. Figure 2A,B show an example the output of a pair of gamma analyses of γ_AC_ and γ_LV_ with results of 100.0% and 99.6%, respectively. Please note that the LinacView analysis, Figure 2B, is rotated 90 from the ArcCheck^®^ analysis. In terms of sensitivity of detecting errors, both systems were able to detect incorrectly matched plan delivery. Figure 2C and D show an example the analysis of an incorrectly matched plan delivery with γ_AC_ and γ_LV_ showing significantly lower gamma passing scores of 84.8% and 21.7%, respectively. The γ (3%, 3 mm) for the 190 correctly delivered fields were found to be in the range of 72.5%–100.0% and 95.4%–100.0% for γ_AC_ and γ_LV_, respectively. Three instances of γ_AC_ were found to be lower than 90% which was attributed to the model deficiency of the couch transmission in MTPS. Excluding these fields, the γ_AC_ range was found to be 90.0%–100.0%. The γ(3%, 3 mm) for the 30 incorrectly matched fields were found to be 3.9%–84.8% and 0.1%–64.4% for γ_AC_ and γ_LV_, respectively. The overall ranked‐correlation between them was found to be 86.0% with *p*‐value <0.01.

**FIGURE 3:**
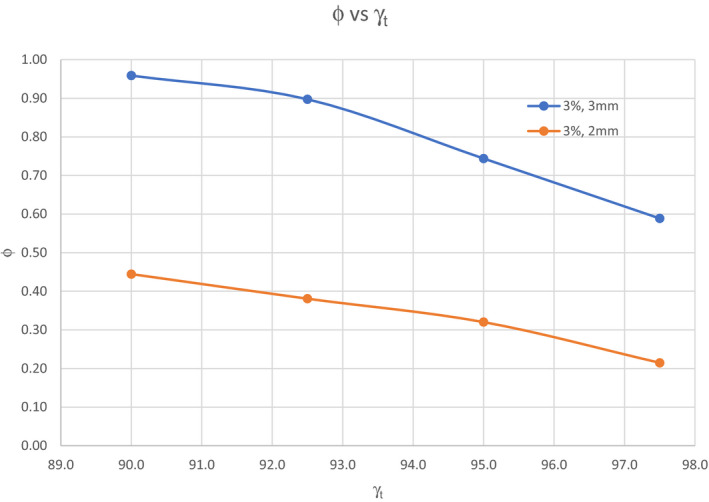
The variation of ϕ with different threshold, γ_t_. Blue line represents γ (3%, 3 mm) and orange line represents γ (3%, 2 mm)

The contingency tests showed the γ_AC_ and γ_LV_ to be significantly associated with ϕ for γ_t_ of 90%–97.5%, respectively. All the ϕ were found to have *p*‐value <0.001. Figure 3 shows the relationship between the ϕ and the γ_t_. In the case of γ (3%, 3 mm) criteria, the ϕ was found to decrease from 0.96 to 0.59 with increasing passing threshold from 90.0% to 97.5%. With γ (3%, 2 mm) criteria, the ϕ decreased from 0.44 to 0.21 with the same passing threshold.

**FIGURE 4:**
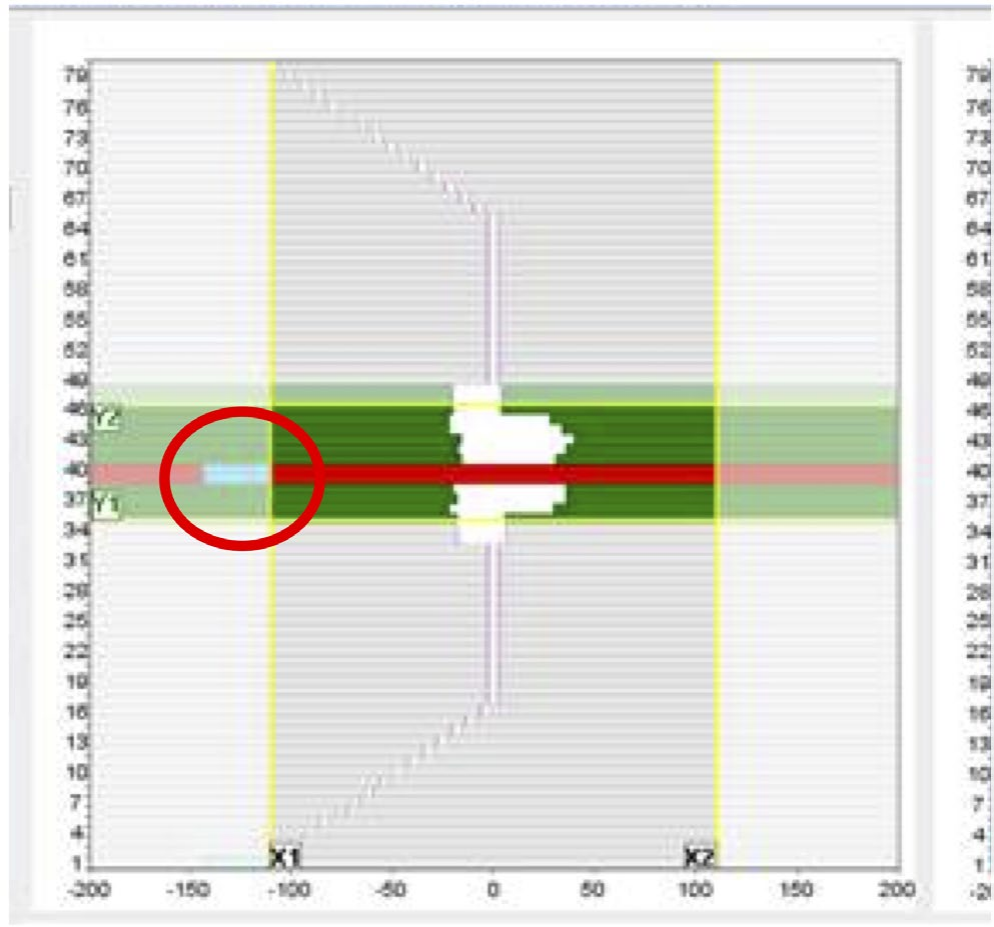
A snapshot of an IMRT step‐and‐shoot delivery that captures number 40 of MLC leaves with more than 30 mm deviation (shown in red circle). Number 40 leaf pair was flagged as failed. This is attributed to the linac MLC controller overriding the treatment plan position

During this study, we noticed the log‐file reported MLC positions significantly different, as much as 30 mm, from the plan positions which could amount to an order of magnitude larger than our institution experience^16^ and other works.^3^, ^6^, ^17^, ^18^ After some investigations, it was found that the MLCs, outside the guard leaves or under the diaphragms, often had more than 1.0 mm discrepancy. Figure 4 shows an example of MLC leaf deviation of more than 30 mm (in red circle). This is attributed to the MLC controller overriding the treatment plan position for closed leaves parked under the diaphragm.^19^ No dosimetry impact on the plan was found. Excluding these discrepancies, all MLC positions were found to be within 1.0 mm. However, because of this issue, MLC position could not be used to correlate with γ_AC_.

## 4 DISCUSSION

Both measurement‐based and log‐file‐based PSQAs play an important role in the overall PSQA process. The strength of each method can complement each other and build a stronger QA program. This combination is particularly essential in meeting the resource intensive online ART program. Although our study found that the overall quality assurance of γ_LV_ has significant correlation with γ_AC,_ complement the other published works,^5^, ^17^ certain discrepancies between γ_AC_ and γ_LV_ were observed which were attributed to unaccounted factors in the log‐file fluence analysis, such as the couch transmission, measurement setup, and treatment planning system accuracy. For example, we found that in 1.5% of all the fields, γ_AC_ was significantly lower than γ_LV_. This was attributed to the couch model in the treatment planning system which could not fully model the transmission accurately^12^ at all gantry angles. This prompted a change in the treatment planning protocol to minimize the use of the gantry angle range of 110–140° and 220–250°. These factors would likely be amplified at higher γ_t_ resulting in lower correlation as observed in this study. A reasonable γ_t_ that is relevant to the clinical practice should be chosen. To reduce the systematic errors stemmed from the couch transmission modeling, a more accurate model in the treatment planning system would be a welcomed improvement.

An investigation was initiated by the MLC discrepancy alerts from the log‐file analysis. Comparing the LogfileSync generated log‐files with the Unity generated log‐files, no discrepancy was found. After discussing with the linac vendor,^19^ it was confirmed that there was a bug with the MLC controller. In this particular MR‐Linac system, the treatment planning system was designed to park all the closed leaves, which were not under the X diaphragm, at 13.0 and 13.4 cm regardless of the positions in the plan. However, the exported RT plan, as a result of MLC controller bug, would show the closed leaves at 11.0 cm. As this issue only affects closed MLC leaves under the diaphragm, the impact in dosimetry would be small and unlikely be caught without the log‐file‐ based QA tool. This demonstrated the added value of increased understanding of the machine operation and its overall value to the QA process. As this MLC controller bug will likely be remedied in the future update, a correction between MLC position and measurement‐based metric can likely be established in the future. At this point, however, the γ_LV_ with the fluence is a better metric to augment the current measurement‐based PSQA.

Ongoing PSQA will be compared with log‐file analysis to investigate the longer‐term reproducibility and correlation. The current system can potentially be developed into a real‐time monitoring system. This will be investigated in future work. The log‐file analysis not only can provide higher throughput due to automation but can also provide faster PSQA feedback to clinicians and physicists.

## CONFLICT OF INTEREST

One of the authors is the employee of Standard Imaging Inc.

## AUTHOR CONTRIBUTION

S. Lim and D.M. Lovelock conceived the presented idea. S. Lim developed the theory and performed the experiment and the computations. M. Napolitano implemented the log‐file listener. P. Godoy Scripes, E. Subashi, and N. Tyagi performed the ArcCheck measurements. S. Lim wrote the manuscript with support from L. Cervino and D.M. Lovelock. All authors discussed the results and contributed to the final manuscript.

## Data Availability

Research data are not shared.
